# Assessment of cognitive function in bipolar disorder with passive smartphone keystroke metadata: a BiAffect digital phenotyping study

**DOI:** 10.3389/fpsyt.2025.1430303

**Published:** 2025-03-06

**Authors:** Olusola Ajilore, John S. Bark, Alexander P. Demos, John Zulueta, Jonathan Stange, Jennifer Duffecy, Faraz Hussain, Scott A. Langenecker, Peter Nelson, Kelly Ryan, Melvin G. McInnis, Alex Leow

**Affiliations:** ^1^ Department of Psychiatry, University of Illinois College of Medicine, Chicago, IL, United States; ^2^ Department of Psychology, University of Illinois Chicago, Chicago, IL, United States; ^3^ Department of Psychology and Psychiatry and the Behavioral Sciences, University of Southern California, Los Angeles, CA, United States; ^4^ Department of Psychiatry, The Ohio State University, Columbus, OH, United States; ^5^ Department of Bioengineering, University of Illinois Chicago, Chicago, IL, United States; ^6^ Department of Psychiatry, University of Michigan, Ann Arbor, MI, United States

**Keywords:** digital biomarkers, bipolar disorder, executive function, smartphone, digital phenotyping

## Abstract

**Background:**

Cognitive dysfunction in bipolar disorder persists in the euthymic state and has been shown to be associated with a number of negative sequelae including treatment resistance and increased risk of relapse. There has been recent attention on digital phenotyping and passive sensing through smart, connected devices to probe cognition in real-world settings. BiAffect is a custom-built smartphone keyboard that captures keystroke metadata (‘how you type, not what you type’). In previous studies, our group has demonstrated that BiAffect-derived keystroke metadata is associated with cognitive domains like processing speed. For the present study, we hypothesized that typing metadata would be significantly associated with executive function and planning.

**Methods:**

18 participants with bipolar disorder and 12 healthy comparison participants from the Prechter Longitudinal Study of Bipolar Disorder at the University of Michigan were provided a mobile phone with a customized keyboard that passively collected keystroke metadata. Participants also completed a neuropsychological battery including the Tower of London task. Irregularities in typing and times to make a move on the Tower of London task were compared using sample and Shannon entropy, respectively.

**Results:**

Participants with bipolar disorder had significant increases in entropy in typing (*p* = .005, *d* = -1.28) and entropy of Tower of London move times (*p* = .029, *d* = -.84). Furthermore, typing entropy was significantly associated with irregularity in Tower of London moves in participants (*r* = .59, *p* = .006), as well as variability of clinician-rated depressive symptoms and self-rated impulsive actions and feelings.

**Conclusions:**

This pilot study demonstrates that passive, unobtrusive smartphone keystroke metadata can be used to probe cognitive function and dysfunction in bipolar disorder, revealing multi-scalar behavioral features accessible through digital assays

## Introduction

Cognitive dysfunction in bipolar disorder has been shown to be present even in the euthymic state (1). Cognitive dysfunction in bipolar disorder has been associated with poor treatment response, increased risk of relapse (2), and comorbid substance abuse (3). In bipolar depressive disorders, patients demonstrate executive function deficits such as impaired response inhibition and difficulties with set-shifting. These cognitive deficits are often associated with illness course and poor treatment response. In addition, the further implications of cognitive dysfunction have been demonstrated in first episode bipolar patients where better performance on frontal/executive tasks was significantly correlated with faster time to recovery (4) and work status (5).

The literature on cognitive dysfunction demonstrates that not only can these deficits occur during an acute episode and impact treatment response but can also occur during euthymic states and predict symptom recurrence and relapse. Furthermore, these deficits broadly have a negative impact of emotion regulation across the spectrum of mood disorders, as well as in patients with bipolar disorder (6–8). Additionally, targeting cognitive dysfunction can augment current treatment modalities and prevent future mood episodes (9). Because of this, monitoring cognitive performance in patients with mood disorders has tremendous clinical implications. However, traditional cognitive assessment can be expensive, time-consuming, and temporally inexact, thus limiting its use and widespread implementation. These limitations demonstrate the need for passive, unobtrusive, objective assessment of cognitive function in the context of mood disorders. There has been recent attention on digital phenotyping and passive sensing through smart, connected devices to probe cognition in real-world settings. Our passive sensing, BiAffect, is a custom-built smartphone keyboard that captures keystroke metadata (‘how you type, not what you type’) (10). In previous studies, our group has demonstrated that BiAffect-derived keystroke metadata is associated with mood symptom severity and can be used prospectively to predict changes in mood in patients with bipolar disorder (11). For the present study, we hypothesized that typing metadata would be significantly associated with executive function measured with traditional cognitive testing in participants with bipolar disorder.

In order to compare typing metadata to traditional cognitive testing, we employed a dynamical systems approach to time-series derived from typing metadata and cognitive performance by utilizing sample entropy analysis (12, 13). Sample entropy analysis has been successfully applied to analyzing time series data from several clinical domains, including but not limited to bipolar disorder to predict changes in mood self-report (4), motor activity (7), as well as motor activity in depression (8), and a range of the other physiological disorders such as heart disease (5). In taking this dynamical systems approach, we were also interested in examining how typing dynamics relates to variability of depressive symptoms and impulsivity, as our previous study demonstrated that instability of mood ratings were associated with worsening depressive symptoms (11).

**Figure 1:**
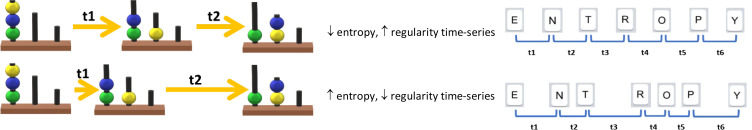
Description of entropy values. T1, T2,…Tn refers to the move time intervals on either of the Tower of London task (left) or interkey delays from keyboard dynamics (right). Higher entropy values are indicative of more irregularity of move times.

For the present study, we hypothesized that if irregularity of move times on the Tower of London task is reflective of poor planning or impulsivity, participants with bipolar disorder will demonstrate higher levels of entropy in move times compared to healthy comparison participants. Furthermore, we hypothesized that higher entropy of move times on the Tower of London task would be associated with higher levels of entropy in typing dynamics for participants with bipolar disorder (**Figure 1**).

## Materials and methods

### Participant recruitment

18 participants with bipolar disorder (12 with bipolar I, 6 with bipolar II) and 12 healthy comparison participants from the Prechter Longitudinal Study of Bipolar Disorder at the University of Michigan (14) completed informed consent in accordance with the Declaration of Helsinki and were provided a mobile phone with the BiAffect app. Information regarding data collection from this cohort have been described in more detail in previous publications (10, 11, 15).

### Cognitive assessment

**Figure 2:**
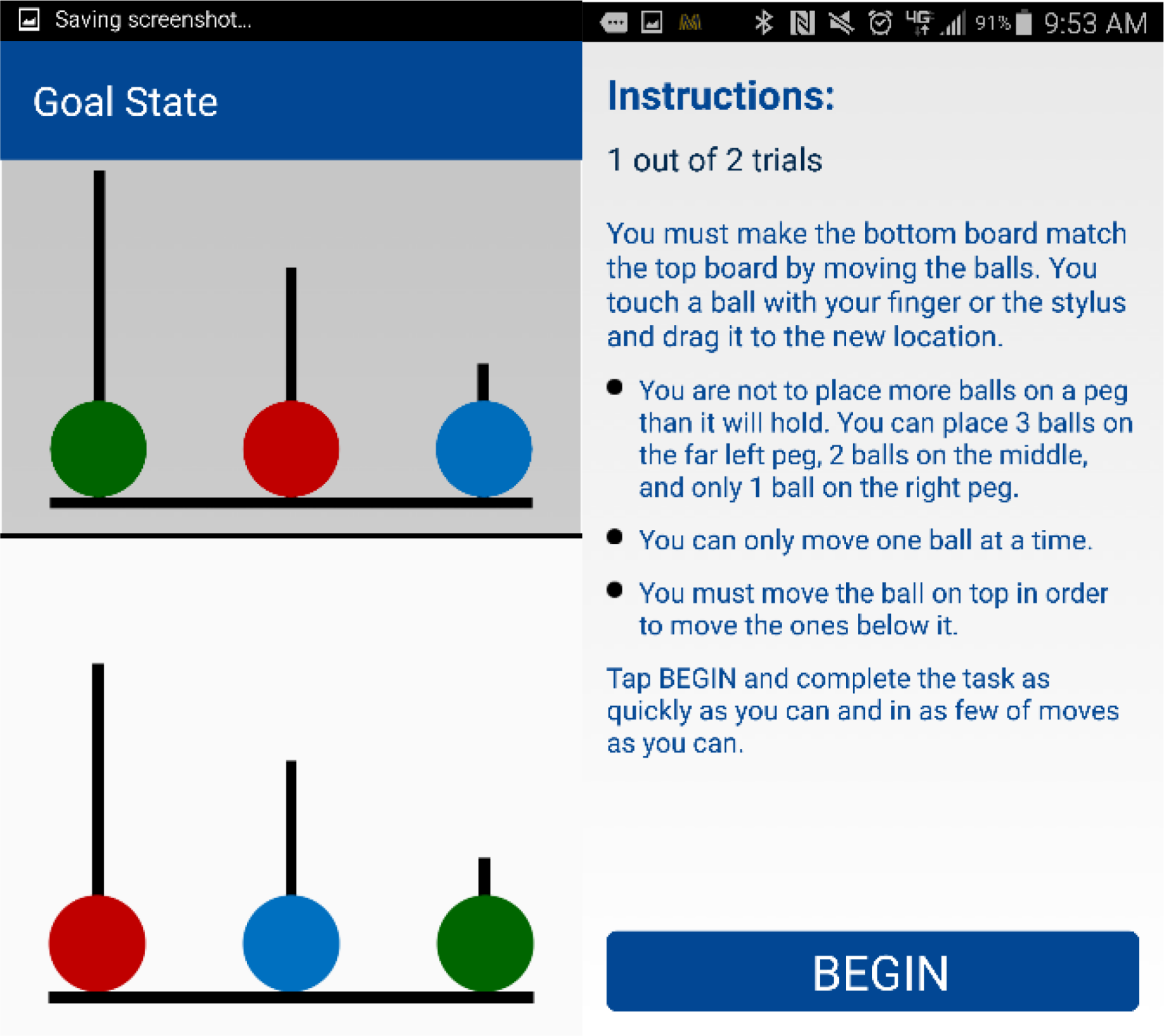
Tower of London task. Instructions that were given before starting the task “You must make the bottom board match the top board by moving the balls. You touch a ball with your finger and drag it to the new location. You are not to place more balls on a peg than it will hold; You can place 3 balls on the far-left peg, 2 balls on the middle, and only 1 ball on the right peg. You can only move one ball at a time. You must move the ball on top in order to move the ones below it. Tap BEGIN and complete the task as quickly as you can and in as few of moves as you can.” If participants did not finish the task within 2 minutes, they received this prompt “That was a good try. Let’s go to the next one.”.

Participants completed a custom made cognitive task on the smartphone modeled after the Tower of London task (16), a test developed to measure planning and problem solving. In the custom smartphone task, participants were to replicate the top configuration of colored, stacked balls by making the bottom pegboard look the same (**Figure 2**). The top pegboard was predetermined (includes multiple versions) and the bottom pegboard is manipulated by the participant. The bottom configuration of balls are touch-sensitive so that the participant can move/drag one ball from one peg and drop/place onto another peg. The pegs vary in height so that the tallest peg on the left can hold up to three balls, the middle can hold up to two balls, and the short peg can only hold one ball. For each trial, there is a preset configuration for the bottom configuration for all moves. There are set rules for moving the balls: 1) can only move one ball at a time; 2) cannot put more balls on a pole than can fit; 3) cannot move a bottom ball without moving the ball on top first. Participants are recorded on how long it takes them to complete the bottom configuration to match the top and the number of moves they make (total time to complete configuration, the time it takes move by move, and number of moves it takes to match the top configuration. The minimum number of moves for each problem was preset at 5 and 6, defined to be a “moderate” difficulty level, each one to be given randomly in the morning ecological momentary assessment (EMA) session and in the evening EMA session. For example, they were given a 5- and a 6-move problem in the morning. We administered two problems so that the task could remain brief (as opposed to up to 30 moves of increasing difficulty, ranging from 10 moves to 32 moves on variant versions of the Tower of London) and include moderate difficulty problems to obtain a range of move times and errors. A total of 11 unique configurations of 5-move problems and 11 unique configurations of 6-move problems were randomly assigned so that no configuration was repeated until all versions were completed, thus minimizing any learning effects. Thus, participants completed a total of 22 problems across all sessions.

### BiAffect and ecological momentary assessment

Participants used BiAffect-equipped Android smartphones and completed a 17-day baseline EMA period during which they were prompted, once daily, to rate their mood, energy, rapidity of thinking, and impulsive feelings and actions using a visual analog scale of 0 to 100 (17, 18). Three participants had less than 17 days collected (11, 15 and 16 days collected). Simultaneously, BiAffect unobtrusively collected typing kinematics metadata as participants interacted with their phones as usual, allowing us to extract average typing speed (measured using time since last key or interkey delay) within each day in their natural environments. Participants also completed a baseline clinician-rated Hamilton Rating Scale for Depression (HAM-D) (19) at study entry. Variability of EMA and HAM-D measures were obtained using the standard deviation.

### Entropy analysis

To calculate entropy for the Tower of London task, Shannon entropy (20) was calculated on move times as a function of trial order across all sessions. Shannon entropy is a measure of uncertainty or randomness in a complex system. Shannon entropy was selected due to Tower of London move times being measured on the order of seconds and the length of the total time-series. For calculation of entropy from typing data, sample entropy (21) was calculated based on the interkey delay as a function of keypress order across all keypresses with interkey delays less than 96 seconds (which represents delays between letters, words, sentences, and conversations).

### Statistical analysis

*t*-tests were used to analyze group differences for continuous variables and the chi-square test was used for categorial variables. Pearson’s correlations were used to analyze the association of typing entropy and Tower of London entropy, as well as typing entropy and self-rated EMA measures.

## Results

### Participant characteristics

Clinical, demographic and Tower of London performance data are summarized in **Table 1**. This cohort was derived from the larger Prechter Longitudinal Study which was 85% Caucasian with bipolar disorder onset at the average age of 17 years (14). There was no significant difference in age, sex, education, or IQ (all p >.05). On the Tower of London task, participants with bipolar disorder took longer to complete the task and had more total moves but these differences were not statistically significant (p = .16 and p = .21, respectively) after controlling for age and education.

**Table 1:** Clinical, cognitive, and demographic participant characteristics.

	Control (n =12)	Bipolar (n=18)	
Age [range]	44.9 (9.0) [30 – 61]	47.4 (10.6) [31 – 63]	t (40) = 1.1 p = .28
Sex (M/F)	4/8	6/12	χ2 = 0.1 P = .92
Education (years)	16 (1.3)	15.8 (1.9)	t(40) = .70 p = .49
WAIS-IQ	108.8 (11.4)	108.5 (7.4)	t(29) = -.05 p = .97
Mean HAM-D	0.7 (0.9)	10.7 (5.4)	t(29) = 6.5 p <.001
TOL duration (seconds)	15.7 (9.0)	18.6 (11.3)	t(2242) = -4.5 p <.001
Total TOL legal moves	7.9 (3.3)	8.4 (3.9)	t(2242) = -2.9 p <.001

All data are represented as means (standard deviations). HAM-D, Hamilton Rating Scale for Depression; TOL, Tower of London.

### Entropy analysis

**Figure 3:**
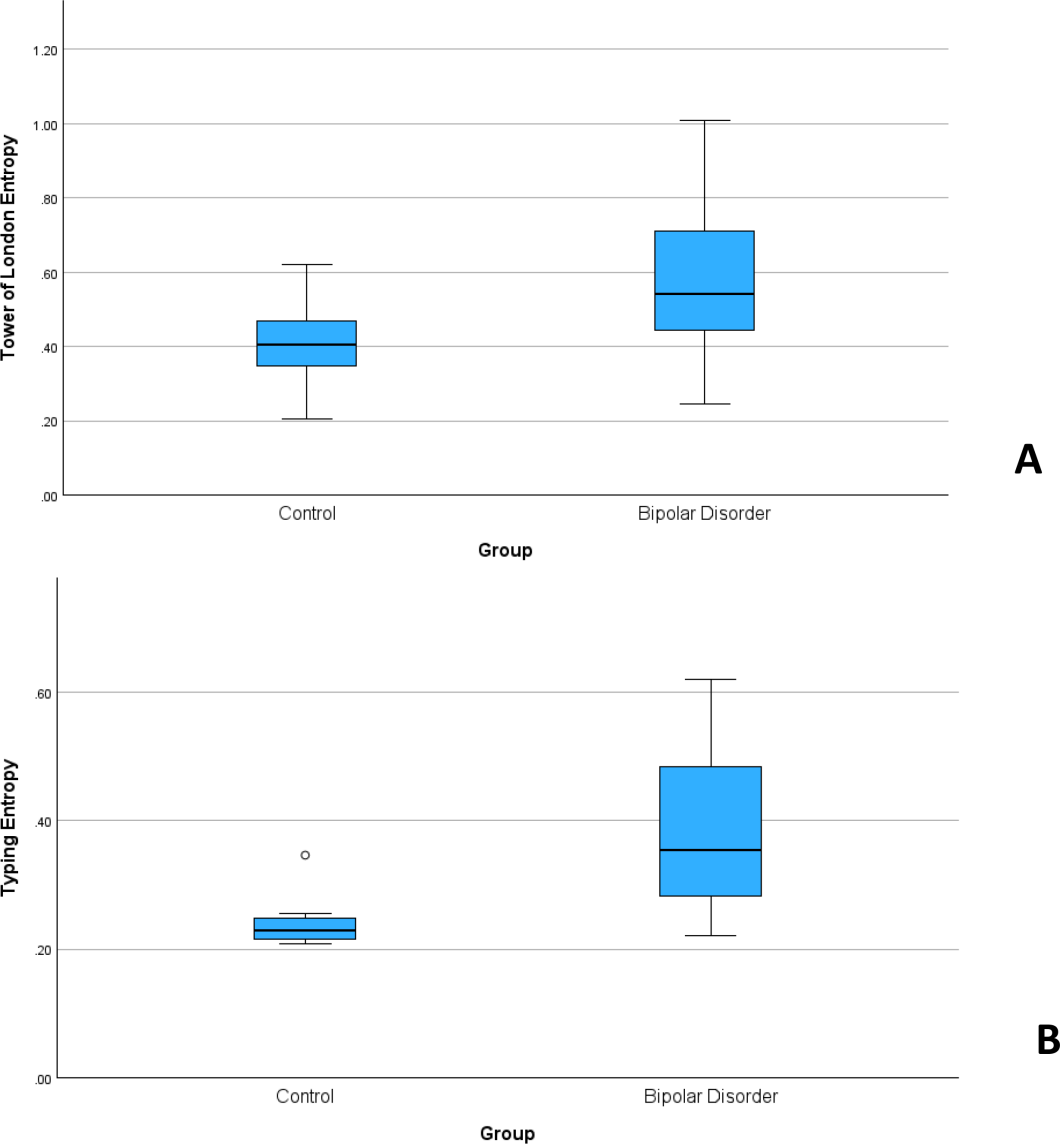
Participants with bipolar disorder had significantly higher levels of entropy as measured from typing **(A)** as well as the performance on the Tower of London task **(B)**.

**Figure 4:**
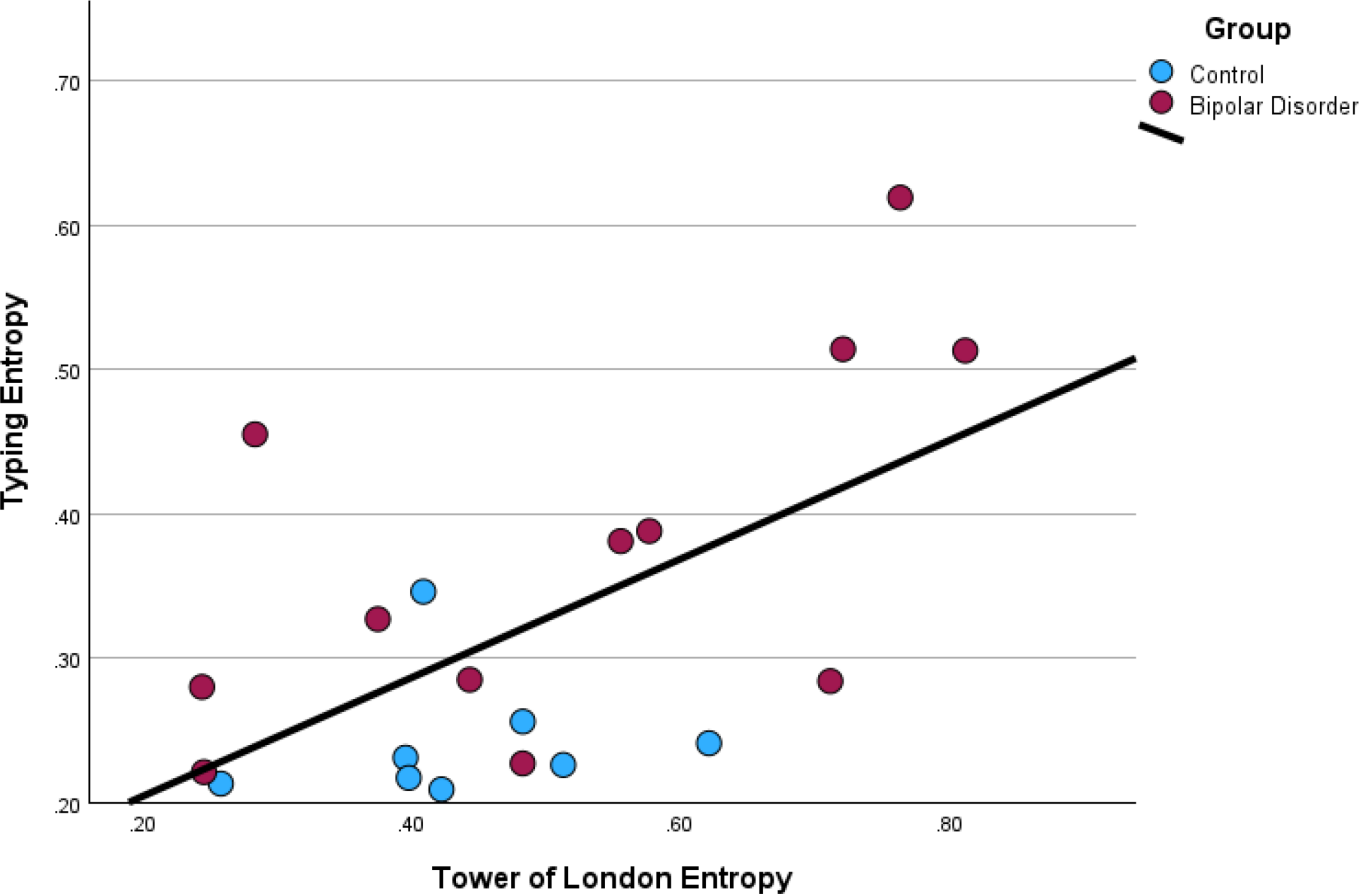
Typing entropy was positively associated with Tower of London entropy.

Participants with bipolar disorder had significant increases in entropy of interkey delay times (typing entropy) (*p* = .005, *d* = -1.28) and entropy of Tower of London move times (*p* = .029, *d* = -.84) (**Figure 3**). Typing entropy was significantly correlated with Tower of London entropy (**Figure 4**, *r* = .59, *p* = .006), variability in depressive symptoms as measured by the HAM-D (*r* = .57, *p* = .009), and variability in self-rated impulsive actions (*r* = .52, *p* =.04) and feelings (r = .59, *p* = .02). There were no significant associations of typing entropy with the average values of EMA ratings of mood, energy, impulsive actions/feelings or racing thoughts. There was a significant association of Tower of London entropy with variability of self-rated impulsive actions (r = .43, p = .03) but no significant associations with variability of impulsive feelings or average levels of impulsive feelings or actions.

## Discussion

In this study, we used an innovative approach leveraging entropy to measure poor planning and impulsivity actively (Tower of London) and passively (typing), associated with cognitive dysfunction in the context of bipolar disorder. We found that Tower of London move time entropy was significantly higher in participants with bipolar disorder compared to controls and this was also reflected in significantly higher multiscale entropy in typing speed in participants with bipolar participants. Typing entropy was also positively associated with variability in depressive symptoms and self-rated impulsivity. While our application of entropy measures to cognitive performance metrics (like move times), our findings are consistent with previous studies that have linked lower entropy in motoric behavior to better planning. Interestingly in a review of research on skilled climbing expertise, the authors found that higher entropy was associated with higher climb times and reduced experience and skill with climbing (22). Similarly, it has been shown that higher entropy on a motor preservation task was associated with worse cognitive performance in adolescents with high-risk for psychosis (23).

With regards to the relationship between typing entropy and variability of mood, Stange et al. demonstrated that instability of typing speed and instability of mood both predicted future elevations of depressive symptoms in this same cohort (11). This highlights the potential of these instability metrics being used prospectively to prevent future mood episodes.

### Implications for emotion regulation

We demonstrated that typing entropy was associated with the instability of self-reported impulsivity and mood symptoms. This is reflective of the relationship between impulsivity and emotion dysregulation seen not only in bipolar disorder but transdiagnostically as well. In a study of participants with borderline personality disorder, it was shown that greater levels of emotional dysregulation were associated with higher entropy of choice patterns on a strategic decision-making task (24). Additionally, in alignment with this paper’s findings, a population-based study by Wen and colleagues demonstrated passive detection of impulsivity using the entropy of the smartphone activities (call logs, battery charging and screen status) (25). They found that motor impulsivity was positively correlated with entropy of the screen checking suggestive of poor planning. Of note, impulsivity is one of the core emotion regulation deficits demonstrated in bipolar disorder (26). With regards to mood symptoms and emotion regulation, it has been shown that variability of cognitive performance was associated with negative affect (27).

### Limitations

Our study was limited by a small sample size and a short study duration. Additionally, the small sample precluded analysis of potential confounders like digital literacy, medication usage and familiarity with the use of smartphone. Another limitation is inherent to mobile cognitive assessments, namely the lack of standardization and control of the testing environment. These potential confounders are being addressed in our ongoing BiAffect studies where we are collecting this important contextual information (ClinicalTrials.Gov NCT04358900).

## Conclusions

In summary, our pilot study demonstrates that passive, unobtrusive smartphone keystroke metadata can be used to probe cognitive function and dysfunction in bipolar disorder, revealing multi-scalar behavioral features accessible through digital assays. Future work is needed in larger samples to validate these findings and to determine whether this type of passive digital biomarker data can be used as an early warning sign to enable just-in-time adaptive interventions and prevent future mood episodes.

## Data Availability

The raw data supporting the conclusions of this article will be made available by the authors, without undue reservation.
